# Adolescent Levers for a Diet and Physical Activity Intervention Across Socioecological Levels in Kenya, South Africa, Cameroon, and Jamaica: Mixed Methods Study Protocol

**DOI:** 10.2196/26739

**Published:** 2021-07-13

**Authors:** Feyisayo A Odunitan-Wayas, Pamela Wadende, Ebele R I Mogo, Anna Brugulat-Panés, Lisa K Micklesfield, Ishtar Govia, Clarisse Mapa-Tassou, Gudani Mukoma, Joanne A Smith, Molebogeng Motlhalhedi, Yves Wasnyo, Vincent Were, Felix Assah, Kufre J Okop, Shane A Norris, Charles Obonyo, Jean Claude Mbanya, Marshall K Tulloch-Reid, Abby C King, Estelle V Lambert, Tolu Oni

**Affiliations:** 1 Health Through Physical Activity, Lifestyle and Sport Research Centre, Division of Exercise Science and Sports Medicine Department of Human Biology, Faculty of Health Sciences University of Cape Town Cape Town South Africa; 2 School of Education and Human Resource Development Kisii University Kisii Kenya; 3 MRC Epidemiology Unit University of Cambridge Cambridge United Kingdom; 4 South African Medical Research Council/Wits Developmental Pathways for Health Research Unit (DPHRU) School of Clinical Medicine, Faculty of Health Sciences University of the Witwatersrand Johannesburg South Africa; 5 Caribbean Institute for Health Research The University of the West Indies Kingston Jamaica; 6 Health of Populations in Transition (HoPiT) Research Group University of Yaoundé I Yaoundé Cameroon; 7 Centre for Global Health Research Kenya Medical Research Institute Kisumu Kenya; 8 Department of Epidemiology & Population Health Stanford University School of Medicine Stanford, CA United States; 9 Department of Medicine Stanford University School of Medicine Stanford, CA United States; 10 Research Initiative for Cities Health and Equity School of Public Health and Family Medicine University of Cape Town Cape Town South Africa

**Keywords:** adolescent, food intake, foodways, physical activity, noncommunicable diseases, socioecological levers, low and middle income countries, health outcomes

## Abstract

**Background:**

The increasing burden of noncommunicable diseases that are prevalent in low- and middle-income countries (LMICs) is largely attributed to modifiable behavioral risk factors such as unhealthy diets and insufficient physical activity (PA). The adolescent stage, defined as 10 to 24 years of age, is an important formative phase of life and offers an opportunity to reduce the risk of noncommunicable diseases across the life course and for future generations.

**Objective:**

The aim of this paper is to describe a protocol for a study using a convergent mixed methods design to explore exposures in the household, neighborhood, school, and the journey from home to school that may influence diet and PA behaviors in adolescents from LMICs.

**Methods:**

Male and female adolescents (n≥150) aged between 13 and 24 years will be recruited from selected high schools or households in project site countries to ensure the socioeconomic diversity of perspectives and experiences at the individual, home, and neighborhood levels. The project will be conducted at 5 sites in 4 countries: Kenya, Cameroon, Jamaica, and South Africa (Cape Town and Johannesburg). Data on anthropometric measures, food intake, and PA knowledge and behavior will be collected using self-report questionnaires. In addition, a small number of learners (n=30-45) from each site will be selected as citizen scientists to capture data (photographs, audio notes, text, and geolocations) on their *lived experiences* in relation to food and PA in their homes, the journey to and from school, and the school and neighborhood environments using a mobile app, and for objective PA measurements. In-depth interviews will be conducted with the citizen scientists and their caregivers to explore household experiences and determinants of food intake and foodways, as well as the PA of household members.

**Results:**

The study described in this protocol paper was primarily funded through a UK National Institute for Health Research grant in 2017 and approved by the relevant institutional ethics review boards in the country sites (South Africa, Cameroun, and Jamaica in 2019, and Kenya in 2020). As of December 23, 2020, we had completed data collection from adolescents (n≥150) in all the country sites, except Kenya, and data collection for the subgroup (n=30-45) is ongoing. Data analysis is ongoing and the output of findings from the study described in this protocol is expected to be published by 2022.

**Conclusions:**

This project protocol contributes to research that focuses on adolescents and the socioecological determinants of food intake and PA in LMIC settings. It includes innovative methodologies to interrogate and map the contexts of these determinants and will generate much-needed data to understand the multilevel system of factors that can be leveraged through upstream and downstream strategies and interventions to improve health outcomes.

**International Registered Report Identifier (IRRID):**

DERR1-10.2196/26739

## Introduction

### Background

There is a rising global burden of noncommunicable diseases (NCDs), resulting in approximately 70% of the deaths, of which more than three-quarters are from low- and middle-income countries (LMICs) [1,2]. A significant proportion of these deaths is attributable to modifiable behavioral risk factors such as poor or unhealthy diets (high consumption of sugar, fats, and salt and low consumption of fruits and vegetables) and insufficient physical activity (PA), both of which are associated with obesity, a metabolic risk factor for NCDs [3]. Of note, the global prevalence of overweight or obesity and physical inactivity is on the rise [1,4,5].

Adolescence, recently defined as 10-24 years of age by Sawyer et al [6] is a *transitory period* accompanied by physical, psychological, and social development and by increasing socialization with peers and independence outside of the family [6,7]. The adolescent period offers an opportunity to reduce intergenerational NCD risk because the increasing independence that characterizes this period of life makes it an important entry point for interventions to promote health across the life course [8]. This age group comprises approximately one-quarter of the world’s population, with most of the adolescents living in LMICs [9]. The global prevalence of obese adolescents in 2016 was more than double the prevalence in 2000 [10] and underscores the current global epidemic of risk factors for NCDs in LMICs [7,11].

Factors such as poverty and the uneven distribution of wealth, lack of education, and urban migration—often into informal settlements—are known contributors to the rising incidence and prevalence of NCDs, especially in LMICs [12]. Consequently, addressing the growing burden of obesity and NCDs effectively requires a focus on different individual and contextual social and environmental exposures and on the interplay among micro-, meso-, and macrolevel factors that influence obesogenic health behaviors [13]. However, it is difficult to intervene in complex urban health realities without taking into consideration the factors that also influence health behavior decisions, many of which go beyond personal choice and include factors at the community, economic, and cultural levels.

Despite these concerns, there is a dearth of research from LMICs on the interplay among the factors at different socioecological levels that influence behavior, especially in adolescents, despite the consensus that this knowledge is important. Consequently, there is an apparent *disconnect* between *lived experiences* and evidence generated to support public health recommendations and policies, with a lack of contextually relevant interventions in these settings [14] and little to no evidence for scalability and sustainability.

**Figure 1:**
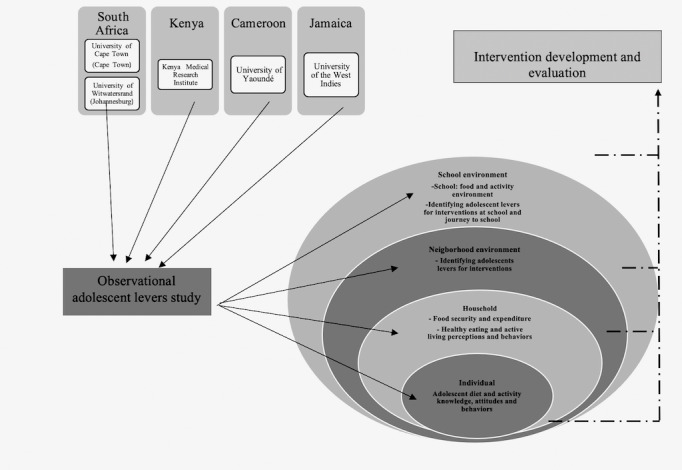
Socioecological framework of the project.

The proposed study described in this protocol paper will use a convergent mixed methods design including survey, observational data, and ethnographic research to identify socioecological influencers of diet and PA in adolescents living in various LMIC settings (Figure 1). This project is 1 work package in a portfolio of projects of the Global Diet and Activity Research (GDAR) network. This network aims to address the previously detailed knowledge gaps, with the overall goal of contributing to the prevention of NCDs in LMICs, with a specific focus on Kenya, Cameroon, South Africa, and Jamaica—countries with moderate to high levels of income disparity [15].

### Objectives

The specific objectives of this project on NCD prevention in LMICs are as follows:

1. To measure dietary behavior (dietary patterns, nutrition knowledge, and nutritional status) and PA behavior (total and domain-specific) in adolescents from low- and middle- to high-income communities.
2. To explore household exposures that may influence dietary and PA behavior in adolescents from low- and middle- to high-income communities.
3. To identify neighborhood-based exposures that may influence dietary and PA behavior in adolescents from low- and middle- to high-income communities.
4. To identify school-based exposures that may influence dietary and PA behavior in adolescents from low- and middle- to high-income communities.
5. To identify exposures that may influence dietary and PA behavior in adolescents on the journey between home and school.
6. To explore the relationships between the socioecological factors that may influence dietary and PA behavior and individual-level factors in adolescents.
7. To explore the similarities and differences among various LMIC settings and across socioecological domains.

## Methods

### Overview

The GDAR network was launched in 2017 with the overall goal of contributing to the prevention of NCDs such as type 2 diabetes, cardiovascular disease, and cancers in LMICs. The GDAR network is funded through a UK National Institute for Health Research (NIHR) grant.

In this paper, we present our original field protocol and describe modifications made following the COVID-19 pandemic, which interrupted data collection at the home-, neighborhood-, and school-environment levels. We describe new approaches that are being taken to meet our study objectives and improve our understanding of the determinants of diet and PA in adolescents.

### Ethical Considerations

We have obtained approval for the study protocol and amendments made because of the COVID-19 pandemic from the University of Cambridge Research Ethics Committee (PRE.2019.105) and local and university ethics committees at the different sites (Cape Town: University of Cape Town Human Research Ethics Committee: 088/2019; Johannesburg: University of the Witwatersrand Human Research Ethics Committee [Medical]: M171137 and M190523; Kenya Medical Research Institute: KEMRI/SERU 4040; Cameroon: Centre Regional Ethics Committee for Human Health Research CE No. 1836 CRERSHC/2020; and the University of the West Indies: ECP 87, 18/19), as well as from relevant educational authorities such as school district management authorities and school principals of selected schools. The study will be conducted in accordance with the Declaration of Helsinki, applying the Society of Adolescent Health Guidelines for Adolescent Health Research pertaining to specific ethical considerations relevant to the protection of minors participating in research [16]. As such, the assent of each adolescent and consent from an adult parent or guardian (for those volunteers under the age of 18 years) will be sought before data collection.

Participant information sheets and consent and assent forms will be translated into the local languages of each country to ensure adequate understanding. All source data will be collected electronically and will be encrypted and password protected. Data-capturing devices will also be password protected, with access limited to the study team. With the exception of the University of the Witwatersrand in Johannesburg, we will recruit participants aged 13 years or older from secondary schools. The University of the Witwatersrand will recruit older adolescents (aged 18-24 years) who have transitioned from high school and will be recruited from an ongoing study in Johannesburg.

Adolescents who participate will be provided with a small noncash reimbursement (such as school stationery or similar items) in remuneration for the time taken to participate. We have provided an option for varying reimbursements to ensure that they could be tailored to each site based on the participating study teams’ knowledge of their country contexts.

### Participant Selection

#### Overview

This protocol paper describes a pilot study; no sample size calculation was conducted because of the lack of baseline data to inform such calculations. It is intended that the collection of several health behavior outcomes across a wide range of settings (and among country sites) will provide information on the variation among groups that will facilitate more accurate sample size calculations for future large-scale research. As such, the sampling size of 150 adolescents from each site allowed us to generate findings across the sites.

#### Cameroon, Jamaica, South Africa (Cape Town)

Adolescents attending school will be categorized using a general sampling guide that defines three categories based on the socioeconomic status of the neighborhood in which their school is located and that of the area where they reside. This is to ensure a diversity of perspectives and experiences. The two socioeconomic categories of the participants’ neighborhoods and schools into low, middle, and high will be determined using a combination of land and property value indices and socioeconomic index map of the locations of their residential areas and schools, respectively, or household assets. The three categories are low-income home neighborhood and low-income school neighborhood (L-L), low-income home neighborhoods and middle- to high-income school neighborhood (L-H), and middle- to high-income home neighborhoods and middle- to high-school neighborhood (H-H), and they are described as follows:

- Adolescents from low-income households attending schools in nearby predominantly low-income neighborhoods (L-L), n≥50 at each site.
- Adolescents from low-income households attending schools farther away in predominantly middle- to high-income neighborhoods (L-H), n≥50 at each site.
- Adolescents from high-income households attending schools in nearby predominantly middle- to high-income neighborhoods (H-H), n≥50 at each site.

Although recruitment of the adolescents will be by convenience sampling, the numbers in each of these three categories will be monitored with the aim of ensuring a roughly equal sex distribution. To prevent any potential for stigmatization, students in middle- to high-income schools who volunteer for participation will be purposively selected according to the residential suburb and nearest street intersection address (L-H and H-H). The basis for the selection will not be shared with the participants beyond the objective of including adolescents who commute to school and that we will aim for geographic representativeness in the sample.

Given the time committed to examinations in the final year of high school and the settling-in period in the first year of high school, this study will target adolescents aged 13 years or older from the second to the penultimate years of high school. As part of the consent process, these adolescents will indicate whether they would be interested in participating in the *citizen scientist* component of the study, which will involve a greater time commitment (Table 1).

**Table 1:** Research activities and objective-specific methodology.

Socioecological domains	Measures	Objectives
Individual (adolescents)	Nutritional knowledge survey Food frequency questionnaire Self-reported PAa tool Anthropometry Demographics Accelerometery (optional)	1, 3, 6, and 7
Household (of citizen scientists)	Household multidimensional poverty index Household demographics and food security Anthropometry (household members-optional) In-depth interviews (foodways and PA)	2, 6, and 7
Neighborhood	Citizen scientists lived experience using EpiCollect5 tool Researchers’ data collection of healthy food and PA opportunities using EpiCollect5 Tool NEWSb Africa (Environmental Walkability) of adolescents- optional	3, 6, and 7
Home-to-school journey (citizen scientists)	Citizen scientists’ lived experience using the EpiCollect5 tool	5, 6, and 7
School	School health environment (policies, facilities, grounds, and resources for healthy eating and PA audit tool) Formal and informal food vendors audit tool	4, 6, and 7

^a^PA: physical activity.

^b^NEWS: Neighborhood Environment Walkability Scale.

#### Kenya

In Kenya, we will follow the same protocol, with one exception. Adolescents from the H-H category will not be included because of logistical challenges inherent in accessing these schools and adolescents (ie, these schools tend to be private schools and are more restrictive about the activities that they allow and parents support on the school premises). As such, adolescents will be recruited in two categories (n≥75 in each category): L-L and L-H (comprising government schools in high-income areas, with a mixture of children from both low- and higher-income households).

The socioeconomic categorization of the participants’ neighborhoods and schools into low and middle to high will be determined using a combination of land and property value indices and socioeconomic index map of the locations of their residential areas and schools, respectively.

#### South Africa (Johannesburg)

In Johannesburg, female adolescents (n≥150) aged 18-24 years who have transitioned out of high school will be recruited from an ongoing larger study, the Healthy Life Trajectories Initiative enumeration platform, which includes individual and household data from more than 2000 households. The Healthy Life Trajectories Initiative platform enables the identification and recruitment of households from low- and high-income settings. Households with adolescents aged 18-24 years will be contacted and invited to participate in the study. The two socioeconomic categories of the participants’ neighborhoods and schools into low, middle, and high will be determined using a combination of land and property value indices and socioeconomic index map of the locations of their residential areas and schools, respectively, or household assets.

### Data Collection

Details of data collection from the different socioecological domains are described herein and summarized in Table 1.

#### Individual Data

Adolescents in schools will have prearranged appointments during or after school, as approved by the school authorities, to participate in the study. Data collection for adolescents who transitioned out of school will be conducted at the research site (South African Medical Research Council/Wits Developmental Pathways for Health Research Unit) in Johannesburg.

#### Questionnaires

Adolescents will be asked to respond to interviewer-administered or supervised self-administered questionnaires (Table 1). The dietary patterns questionnaire will be adapted for each context from the International Study of Childhood Obesity, Lifestyle and the Environment study, a multicountry study of children from 12 countries in 6 continents [17,18]. The nutrition knowledge questionnaire will be adapted from two previously validated instruments [19,20] because it has not been used in these contexts previously. Internal consistency will be assessed using interitem analysis. The PA questionnaire will be adapted from an instrument that has successfully been used in South African adolescents from low-income and rural settings, and it has been validated against objectively measured PA [21].

#### Anthropometric Measurements

Height and weight will be measured using standardized procedures [22] in a private room by a team of uniformly trained research assistants to minimize the effect of interobserver variation, using a stadiometer and calibrated scale, respectively.

Waist circumference will be measured with a nonelastic fiberglass retractable anthropometric tape following standard procedures for measurement [22]. This process will be repeated, and the average of the two circumferences will be used in the analysis (with a third measurement obtained if the first two measurements are more than 0.5 cm apart).

#### Household Data

Household-level data (quantitative and qualitative) will be collected from a subset of adolescents (citizen scientists) and their households as described herein. Before this, pilot interviews using the qualitative tools were conducted at all sites.

#### Objective PA Measurements

In a subset of adolescents (citizen scientists) for the sites that conduct this aspect of the study, daily PA will be measured using small motion sensors known as accelerometers (Actigraph GTX3+ [ActiGraph, LLC] or Axivity [Axivity Ltd] monitors). These devices—all smaller than a matchbox—will be worn on a belt around the waist or on the wrist. The participants will be asked to wear these monitors continuously for a week and remove them only when bathing, showering, or swimming. Data collected with at least 10 hours of uninterrupted wear time for at least 4 days will be considered complete. Nonwear periods are defined as any period of at least 30 minutes of continuous zero counts. We used the World Health Organization criteria for sedentary time and moderate- and vigorous-intensity PA as follows: sedentary time: time spent sitting or lying down with low energy expenditure, such as when watching TV or sitting in class; moderate activity: activity that moderately raises the heart rate, such as walking and performing household chores during the week; and vigorous activity: vigorous heart rate–raising activity during the week. Time spent in sedentary activity and moderate- and vigorous-intensity PA will be determined along with the PA levels in different domains. Given the arbitrary nature of the accelerometer count–based cut-offs and the difference in unit expression across accelerometer models, we will report accelerometry data in standardized units of acceleration (m/s^2^) [23,24].

#### Household Socioeconomic Demographic and Anthropometric Measurements

The head of the household or a member of the household nominated by the adolescent citizen scientist will be invited to participate in the study after which a household survey will be completed. This consists of a multidimensional poverty index tool, will be used to capture household access to services and assets, physical structure of the home, household demographics, education, social grants, employment status, and the Food Insecurity Experience Scale [25] to capture household food security. This survey will be completed during the initial household visit.

During this visit, all household members who have provided consent, including children (aged above 5 years) who can assent, will have anthropometric measurements taken using the same standardized methods as used for adolescent measurement.

#### In-depth Interview on Household Foodways

Trained research team members will conduct semistructured, in-depth interviews with the previously consented primary adult caregiver about the factors that influence the diet and PA behavior of members of the household. This interview will include guiding questions concerning *foodways* [26], including food procurement, storage and preparation, food choices and challenges, leisure time activities, and the meaning and significance of food and PA within families.

#### Neighborhood and Journey-to-School Data

Lived experiences and perceptions of their neighborhoods will be completed by the adolescents using two different tools as follows:

### Neighborhood Walkability Using Neighborhood Environment Walkability Scale-Africa Questionnaire

The Neighborhood Environment Walkability Scale-Africa will be used to capture participant perceptions of the conduciveness of their built environment to walking and similar physical activities. This tool has been validated in several sub-Saharan African countries, including Kenya, Cameroon, and South Africa [27].

### Adolescent Lived Experience Using the EpiCollect5 Tool

A subset of the 150 adolescents recruited will be invited to participate in the *citizen scientist* components of the project (objectives 3 and 5) to collect data within their neighborhood and on their journey to and from school using the EpiCollect5 tool developed by the Centre for Genomic Pathogen Surveillance as part of the Big Data Institute at Oxford University (a description has been provided later in this section). This subsample of adolescents (citizen scientists) will specifically include those self-identified as peer leaders (in school club leadership positions), in addition to general students. Approximately 30-45 adolescents will be recruited from each site as follows: 10-15 adolescents will be recruited from schools in low-income settings (L-L category), and 15-30 adolescents will be recruited from each site in higher-income settings (a mix of adolescents from the L-H and H-H categories detailed previously).

Citizen science is a multidimensional approach to research that considers the challenges experienced by different communities as well as the cultural diversity and differences among the residents. It involves members of these communities and the broader public working with scientists to collect and analyze data [28]. This approach has been used to promote and increase PA and other healthy behaviors [29]. Although it has been used mostly in developed countries, the citizen science approach has been used to determine the enablers and barriers of PA in adults from a low-income urban area in South Africa [30] and in adults and adolescents in Mexico [31].

The citizen scientists will be trained to use the EpiCollect5 tool [32], a simple mobile app, to map and describe their journey to and from school and neighborhood-level factors that affect their diet and PA (*the walk*). The mobile app data consist of geotagged *photovoice* or voice notes, photographs, and global positioning to confirm their location within the community setting. The adolescents will have a list of guiding instructions and questions for these walks. Some of the information they will be asked to capture include the following: food outlets in the area; food and drink advertisements on radio or television; locations at which they buy or are given food and drink; other food options on offer at such locations; locations at which they do not eat or spend time and why; and the factors that hinder or facilitate their PA, such as gym facilities, fields, and so on.

After the EpiCollect5 data collection is complete, to gain further insights into the adolescent lived experience, we will convene focus group discussion workshops with the participants, face to face or virtually, to review their experience as citizen scientists and their observations of their food and PA environments. The citizen scientists will be provided with deidentified photographs and associated narratives of the various EpiCollect5 tool records. In small groups, they will be trained to sort through the various photographs and identify themes. Once themes have been identified, the group will be asked to prioritize those factors that may be barriers to, or facilitators of, healthy food choices and PA opportunities and, more specifically, those that may be able to be changed through advocacy or by engaging with other factors [33]. In addition, as part of the knowledge exchange activities, we will train selected citizen scientists in advocacy and presentation skills to present their findings in collaboration with researchers in a virtual or face-to-face meeting at the end of the data collection phase. Adolescents, school stakeholders, and food vendors around the schools will be invited to this meeting, the purpose of which would be to share results from the study and to hear from these actors on their different roles in contributing to the food and PA environments and possible interventions to make these healthier.

### Researcher Measurement of Healthy Food and PA Opportunities Using the EpiCollect5 Tool

Researchers will use the same EpiCollect5 tool to provide observational measures of access to healthy affordable food and the attributes of the built environment that promote or create a barrier to PA in the domains of transport, sport, or leisure. This will involve geocoding the various amenities, including food outlets (retail, spazas or informal traders and vendors, fast food outlets, restaurants, and so on), PA sites (parks, gyms, and transport hubs within a 1000-m buffer of households), advertising of fast food and sugar-sweetened beverages, and community assets within a 1000-m buffer of each participant’s residence. To account for daily variability in the food and PA environments, data collection will be conducted on 2 separate days when the environments might be expected to change, for example, on weekdays and weekends or on market and nonmarket days.

Recordings will be transcribed, existing nutritional information will be obtained for foods where possible (eg, from manufacturers’ websites), and the foods will be categorized (1) by food group and (2) as more or less healthy.

### School Environment Data

In consultation with the school leadership, a teacher liaison will be recruited as the school *point person*. After obtaining consent, the teacher liaison will complete an interviewer-administered questionnaire and observational checklist on food- and activity-related school policies and on the school diet and PA environments, as detailed herein.

#### Audit of School Policies

An audit of the school policies related to diet and PA will be conducted using an observational checklist that will be completed by study staff combined with a questionnaire completed by the teacher liaison in the school. This will include school policies on physical education and healthy eating.

#### Audit of School Environment

An audit of the built environment in and around selected schools will be completed by the study staff using an observational checklist. This will be combined with a questionnaire completed by the teacher liaison. The audit will assess transport and traffic around the schools and places inside the school grounds to engage in PA (play). The study staff will systematically walk around in the immediate area surrounding the schools (1000-m radial buffer), coding the number, typology, and locations of formal and informal vendors and noting the presence of food shops and vending machines on and around the school grounds.

#### Audit of Food Vendors Around Schools

Additional information will be collected on informal vendors situated around the selected schools and the types of food and beverage items they sell. These assessments will be repeated on different days and at different times of the day to assess the reliability of the observation tool and variability in the environment. All data will be collected using the EpiCollect5 tool, which allows for the electronic capture of audio, photographic, and text data. Voice recordings will be used to describe informal vendors and the food and beverages on offer, using approaches developed and tested in previous studies [34,35]. We will collect information on aspects such as (1) type of food outlet, (2) kind of food outlet (formal or informal), (3) brands and types of food and beverages sold, (4) most frequently purchased food items, (5) prices of healthy and unhealthy foods, and (6) strategies adopted for sales promotion.

#### Protocol Adaptation Because of the COVID-19 Pandemic

Although the aim and objectives of the study remain unchanged, the current context of the global COVID-19 pandemic necessitated some adaptations of research activities, given that data collection was incomplete when lockdown restrictions were implemented at the GDAR partner country research sites. Amendments to specific methods to achieve some of the objectives of the protocol became essential because of movement restrictions at the GDAR partner country sites. These amendments are imperative to capture the levers associated with the diet and PA of citizen scientists and their households before and during the pandemic efficiently and cost-effectively, while simultaneously ensuring the safety of the participants and researchers. Furthermore, these amendments take account of the *new normal* circumstances such as mandatory social distancing and total or partial lockdown of countries, both of which have been shown to affect the food and built environments [36]. The amendments made that were relevant to the data collection methods and data analysis in objectives 2, 3, and 5 are as follows:

Objective 2: Explore household exposures that may influence diet and PA behavior in adolescents from low-to higher-middle–income communities.

The original protocol required face-to-face interviews with the parent or caregiver of the citizen scientist. This method will be revised to include conducting phone interviews with the parent or caregiver, and the guiding questions will be modified to include questions concerning the changes to their diet and PA and other related factors as a result of the COVID-19 pandemic. For example, “Are there any changes to the diet and physical activity of your household during the COVID-19 crisis compared with pre-COVID?”

Objective 3: Identify neighborhood-based exposures that may influence diet and PA behavior in adolescents, from low- to higher-middle–income communities.

Objective 5: Identify exposures that may influence diet and PA behavior in adolescents on the journey between home and school.

According to the original protocol, the citizen scientists and research team members were to conduct *walk-along interviews* in their neighborhood using the EpiCollect5 mobile app to achieve objectives 3 and 5. Thereafter, the citizen scientists by themselves were to capture their journey to school and school lived experiences using the mobile app. These activities might not be feasible as planned because of safety reasons and COVID-19 restriction measures that are in place at the GDAR partner country sites. Consequently, phone interviews with citizen scientists about their *lived experience* and factors that shape or influence their diet and PA behaviors before and during the COVID-19 pandemic will be conducted.

#### Analysis Plan

##### Quantitative Analysis

Multivariable regression analyses examining the associations between the school and home environments and dietary patterns, nutritional knowledge, consumption of specific food groups such as fruit and vegetables, BMI, total PA, and domains of PA will be conducted. We will add interaction terms to explore the distribution of environment-behavior associations by country, socioeconomic area, and socioeconomic status of the adolescents’ households.

##### Qualitative Analysis

Data preparation for analyses will follow the standard guidelines for qualitative research strategies [37]. The voice notes of the citizen scientists’ focus group discussions (workshop) and household in-depth interviews will be transcribed verbatim. Each citizen scientist’s voice note and transcription will be linked to its other relevant files (ie, an electronic version of the focus group seating map, notes on nonverbal communication and other issues observed, typed memos, typed notes from the debriefing session, and a list of actual vs scheduled participants). The participants’ anonymity and confidentiality will be protected for all reporting purposes. The recordings will be kept electronically in password-secured files for 10 years before they are deleted.

We will focus on thematic data analyses. In line with standard practice [38], data analysis will be conducted on an ongoing basis that will begin with the first interviews scheduled and focus groups conducted and will continue throughout the study. Each moderator, notetaker, and research assistant will record in a reflective manner their thoughts about each focus group session, individual interview interactions, and community observations. They will pay special attention to ideas and issues discussed, similarities and differences among the focus groups and individual interviews conducted, things to keep in mind for subsequent contacts, and possible questions for future interactions. During data collection, the investigators and research assistants will meet several times to discuss their findings and identify emerging ideas and topics.

The next step of data analysis will occur when data collection is complete. After the transcription of the qualitative data has been completed, the codebook will be developed inductively, focusing on the objectives of the study and the data collected. Using procedures consistent with textual and content analysis, the focus group and interview data collection personnel and members of the research team will review the transcripts. Each transcript will be read several times and coded line by line. We will use Excel (Microsoft Corporation) and NVivo (QSR International) to highlight words or sentences that capture the critical issues and thoughts identified by the participants. These central categories identified within the material will also be documented according to the preidentified research questions. This process will help us to identify the connections among the categories as part of the clustering process in qualitative analyses. The clusters of categories will facilitate the identification of themes within the data. These themes will then be grouped according to the different health behaviors that are the focus of the qualitative work (engaging in citizen science, food, and PA environment). Deviant cases will also be discussed.

The use of different interviewers, focus group moderators, and notetakers that we aim to have in this study should decrease the likelihood that the findings emerging from each of these data collection modes are a result of personal bias or leading questions. In addition, a review of the transcripts by additional research team members will be conducted to ensure that the findings are grounded in the data.

#### Data Management

Assessment standardization and data quality assurance and fidelity will be optimized through centralized training and oversight of the data collection sites to ensure appropriate handling of sensitive information, uniformity in data collection, data entry, and data quality. Any data resources containing personal health information or otherwise potentially identifiable information will use industry-standard encryption such as Advanced Encryption Standard-256.

For the quantitative data (data security), Advanced Encryption Standard-256 encryption will follow the survey data through its life cycle, when at rest or in transit. Data will be collected and transmitted regularly to the REDCap (Research Electronic Data Capture) data server. The REDCap data server is a Health Insurance Portability and Accountability Act–compliant, firewalled environment with daily automated onsite and offsite backups. The data server is continuously monitored for suspicious intrusion activity.

EpiCollect5 is part of the Big Data Institute at Oxford University and hosted at a UK data center with cloud hosting provider, Digital Ocean. Digital Ocean is fully General Data Protection Regulation compliant and accredited at Cloud Security Alliance STAR Level 1. To prevent unauthorized access and for secure data storage, mobile devices (Android [Google LLC] and iOS [Apple Inc]) will be encrypted and password or fingerprint protected. Data are transferred from mobile devices to the application server using Hypertext Transfer Protocol Secure, and the Transport Layer Security certificate uses SHA-256 with RSA (Rivest-Shamir-Adleman) encryption as a signature algorithm.

Transcripts from qualitative data will be pseudoanonymized (removing direct mention of identifiers) during the quality checks by the researchers after transcription, and audio recordings will be deleted after transcription and quality checks. The only information kept and linked to participant IDs will be broad demographic and role descriptors. Anonymized transcripts will be uploaded to NVivo, an encrypted web-based qualitative analysis software.

Data will be stored, as described previously, at each collaborating country site and transferred between sites through the Secure File Transfer Protocol software set up by the University of Cambridge.

#### Researcher Debrief or Reflection

As described in the analysis plan for objectives 3 and 5, a key qualitative strategy will be memo taking and discussion among the team members of the research activities. These will be ongoing throughout the fieldwork and will be documented in the form of typed notes. These notes and other observations will be discussed at meetings of the data collection staff to document shared and distinct observations of the fieldwork. These data will be analyzed using the techniques described in the analysis plan.

## Results

The primary objective and outcome of the proposed study described in this protocol paper is to determine the barriers and facilitators (levers) of healthy diet and PA of adolescents in their household, neighborhood, and school environments and during the journey from home to school. It is also to compare the similarities and differences of these levers among settings and across socioecological domains. The secondary outcomes include exploring the potential of a participatory citizen science approach to build agency among adolescents to inform future policy to promote a healthy diet and PA. The study described in this protocol was primarily funded through a UK NIHR grant in 2017 and approved by the relevant institutional ethics review boards in the country sites (South Africa, Cameroun, and Jamaica in 2019, and Kenya in 2020). As of December 23, 2020, we had completed data collection from adolescents (n≥150) in all the country sites, except Kenya, and data collection for the subgroup (n=30-45) is ongoing. Data analysis is ongoing and the outputs of findings from the study described in this protocol are expected to be published by 2022. The study timeline is detailed in Table 2.

**Table 2:** Timeline of the study.

Activity		2019	January-March 2020	April-June 2020	July-September 2020	October-December 2020	January-March 2021	April-May 2021	June-December 2021
**Preparation**									
	Ethics submission	✓^a^	✓						
	Initial school enquiries	✓	✓						
	Staff recruitment	✓	✓						
	Standard operating procedures: anthropometrics and data collection tool development and adaptation	✓							
	Identify schools	✓	✓						
	Staff training		✓						
	Pilot tools		✓						
**Data collection**									
	Adolescent recruitment		✓	✓	✓				
	Individual adolescent data collection		✓	✓	✓	✓	✓		
	Household data collection				✓	✓	✓		
	Neighborhood and journey-to-school data				✓	✓	✓		
	School environment data				✓	✓	✓		
**Data analysis**									
	Data analysis						✓	✓	✓
	Results synthesis workshop						✓		
**Write-up of outputs**									
	Write-up and dissemination of outputs							✓	✓

^a^Activity is performed during the time period.

## Discussion

### Importance of This Protocol Paper

This protocol paper for our study describes an investigation into the potential levers that influence adolescent diet and PA in their home, neighborhood, and school environments, as well as in the dynamic exposures that adolescents experience on their routine journey between home and school. We aim to explore the interactions between individual-level factors and these upstream determinants in various socioeconomic groups. The adolescent life-stage offers an opportunity to improve adolescents’ lifestyle behaviors for future generations, which may also lessen intergenerational NCD risk and burden. We envisage that by engaging in *citizen science*, adolescents will develop individual and collective self-efficacy such that they can identify barriers to affordable healthy eating and PA and proffer solutions. We further anticipate that this approach will serve to engage, empower, and connect school-aged *citizen scientists* as catalysts for change in their communities.

The results from this study will feed into policy round table engagement activities to be conducted as part of the broader GDAR research portfolio to facilitate sharing of data with policy makers and stakeholders and inform the co-design of interventions in the built environment that support healthy eating and active living behaviors.

Beyond this study, we hope that participation in this type of research will encourage and inspire adolescents to explore further the possibility of becoming lifelong social catalysts for positive change in their own communities and beyond. By training citizen scientists in low-resource settings to gather evidence, analyze, and disseminate the findings, the community may be able to advance solutions to the barriers to active living and healthy eating, often outside of the health sector.

### Strengths and Limitations

The main strength of this protocol is its novel approach, which triangulates convergent mixed methods design, including survey, observational, and ethnographic data, with citizen science research. This protocol is designed to better understand the unique sociocultural, environmental, ecological, and policy levers that may contribute to effective and sustainable interventions. The study also encompasses levers across socioecological levels (individual, home, school, journey to and from school, and the neighborhood) that could affect the diet and PA of adolescents. Another strength of the proposed research protocol is that it is designed to empower adolescents to develop advocacy skills and individual and collective self-efficacy such that they will be able to engage local *actors* and stakeholders to address the barriers to affordable or accessible healthy eating and PA.

This study is exploratory and hypothesis generating in nature, and it is intended that the collection of a number of health behavior outcomes across a wide range of settings (and across and among country sites) will provide information on the differences and variations among groups, thus informing subsequent hypothesis testing in larger-scale research. The study limitations include the fact that the study being described will be conducted in only 1 city in each of the countries, except for South Africa with 2 cities, but with participants from different age groups, which may limit the generalizability of the findings to other regions within these countries. Furthermore, data will be collected from 1 to 3 schools from different socioeconomic areas in each city, which may not be representative of the different categories of adolescents in our study. The age group of high school adolescents included in the study may not be representative of the entire spectrum of adolescence, particularly the earlier phase of this life period. The analysis plan does not explicitly take into account the ethnicity of the participants, which might have an impact on their diet and PA, particularly in South Africa where ethnicity is more heterogeneous. However, although this is not included in the proposed cross-country comparative analysis because of the relative racial homogeneity in other countries, this can be explored further in a South African in-country analysis.

On the basis of the insights that are achieved through the study, expanding subsequent research to a broader number of cities and more representative adolescent age groups may be indicated.

### Conclusions

The development of research with a focus on the socioecological determinants of diet and PA in LMIC settings and the employment of innovative methodologies to interrogate and map the contexts of these determinants will generate much-needed data to understand the levers that can be leveraged to improve health outcomes. This protocol details a mixed methods study to explore potential levers for intervention to improve adolescent diet and PA at varying socioecological levels from the home and neighborhood to the school environments and the dynamic exposures between home and school. The inclusion in the sample of caregivers and community members who play key roles in decision making about diet and PA is expected to add to the comprehensive nature of the data and the inferences that would be drawn from them. The individual and public involvement processes used will support the development of awareness and advocacy skills among adolescents, which are important for peer-led dissemination of information about NCD risk factors and advocacy for healthier food and built environments to reduce the risk of experiencing NCDs in adolescence and subsequent adulthood.

## Abbreviations

GDAR: Global Diet and Activity Research
H-H: middle- to high-income home neighborhoods and middle- to high-school neighborhood
L-H: low-income home neighborhoods and middle- to high-income school neighborhood
L-L: low-income home neighborhood and low-income school neighborhood
LMIC: low- and middle-income country
NCD: noncommunicable disease
NIHR: National Institute for Health Research
PA: physical activity
REDCap: Research Electronic Data Capture
RSA: Rivest-Shamir-Adleman
